# Platonic Projection Structures: Operator-Induced Observability in Representation Learning

**DOI:** 10.3390/e28070768

**Published:** 2026-07-05

**Authors:** Kazuo Ishii, Bishnu Prasad Gautam, Jieling Wu, Saher Javaid

**Affiliations:** 1 Department of Applied Information Engineering, Faculty of Engineering, Suwa University of Science, Chino 391-0292, Nagano, Japan; gautam_bishnu@rs.sus.ac.jp (B.P.G.); wu_jieling@rs.sus.ac.jp (J.W.); 2 Department of Information Engineering, Kanazawa Gakuin University, Kanazawa 920-1392, Ishikawa, Japan; saher@kanazawa-gu.ac.jp

**Keywords:** observability, representation learning, operator-theoretic framework, positive semidefinite operators, knowledge distillation, interpretability, quotient geometry, quantum measurement

## Abstract

We characterize observability in representation learning through Platonic Projection Structures (PPS), an operator-theoretic framework for analyzing representation accessibility under partial observation. Rather than treating observable outputs as direct reflections of latent representations, PPS models observation as a geometry induced by a self-adjoint positive semidefinite operator acting on a latent Hilbert space. A system is represented as a triple (H,Π,O), where H denotes a latent representation space, Π⪰0 is an observation operator, and O(v)=〈v,Πv〉 defines an induced scalar observable. The framework characterizes observability through the quotient geometry H/ker(Π), which represents equivalence classes of latent states that are indistinguishable under observation. From this perspective, observable behavior is governed not by latent representations themselves, but by the geometry induced through the observation operator. We show that both quantum measurement and representation inference under linear observation models can be formulated within this common operator-theoretic structure while differing in the algebraic properties of their observation operators. Within this perspective, quantum measurement serves primarily as a mathematically canonical example of projection-mediated observability. The correspondence developed in PPS is therefore structural rather than physical. Within the same framework, representation transfer and knowledge distillation can be interpreted as approximate preservation of observable geometry through the intertwining condition $\Phi \Pi_{T}\approx \Pi_{S}\Phi .$ PPS further reveals a structural limitation of output-based interpretability: latent components contained in ker(Π) are fundamentally inaccessible from observables generated through the induced observation process. Accordingly, attribution and explanation methods inherit intrinsic constraints imposed by the observation geometry itself. We provide controlled empirical validations demonstrating kernel-invariant observability, projection-induced attribution gaps, and rank-controlled observable geometry in latent representation spaces. Overall, PPS provides a mathematically explicit characterization of observability through operator-induced quotient geometry, offering a unified perspective on representation accessibility, interpretability, and representation transfer.

## 1. Introduction

Modern machine learning systems are increasingly interpreted through observable quantities such as output distributions, attribution maps, intermediate representations, and feature activations [1,2,3,4,5,6,7]. At the same time, recent developments in information-theoretic analysis have highlighted that observable outputs often provide only partial access to the underlying latent structure of a system [8,9,10,11]. Despite substantial progress in explainable artificial intelligence (XAI), representation analysis, and information bottleneck approaches [1,2,3,8,9,10,11], the notion of *observability* itself remains largely implicit rather than explicitly formalized in modern learning systems. In many existing formulations, observable outputs are implicitly treated as sufficiently informative reflections of latent representations. However, practical learning systems frequently operate under restricted observation geometries in which only selected latent directions contribute to observable behavior. This paper introduces *Platonic Projection Structures* (PPS), an operator-theoretic framework for analyzing projection-mediated observability in representation learning systems [12,13,14]. Rather than treating observation as direct access to latent states, PPS models observable quantities as induced through a self-adjoint positive semidefinite operator acting on a latent Hilbert space [12,14]. Within PPS, a system is represented as

(1) (H,Π,O),

where H denotes a latent representation space, Π⪰0 is an observation operator, and the observable quantity is defined by

(2) O(v)=〈v,Πv〉.

The primary observable object in PPS is the projected representation Πv , which determines observable equivalence classes. The scalar quantity O(v)=〈v,Πv〉 is introduced as an operator-induced energy functional that summarizes certain observable aspects of the representation, but does not by itself characterize the full observable geometry. Observable accessibility is determined by the projection operator through the equivalence relation induced by Π and the resulting quotient structure H/ker(Π) . The central principle of PPS is that observability is governed not by latent representations themselves, but by the geometry induced through the observation operator. These assumptions are standard in operator theory and positive semidefinite analysis [13,14].

The operator Π induces the equivalence relation

(3) $$v_{1}{∼}_{\Pi }v_{2}\;⟺\;v_{1}-v_{2}\in \ker\left(\Pi \right),$$

yielding the quotient structure

(4) H/ker(Π),

which represents the effective observable space. Under this formulation, latent components contained in ker(Π) are structurally inaccessible from observables generated through Equation (2). This viewpoint leads naturally to a spectral characterization of observability through the eigenspectrum of the induced observation operator [15,16,17,18,19]. The eigenspectrum of Π determines which latent directions are strongly observable, weakly observable, or entirely suppressed. Consequently, within the PPS formulation, the effective observable dimension is characterized by the rank and spectral structure of the observation operator [15,18,20]. The PPS framework provides a unified abstraction for projection-mediated observation across multiple domains. This perspective is related to operator-theoretic formulations appearing in quantum mechanics, kernel methods, and information geometry [20,21,22,23,24,25,26]. In quantum systems, idealized measurements correspond to orthogonal projections satisfying [21,22]

(5) $$\Pi^{2}=\Pi ,$$

whereas deep learning systems generally induce non-idempotent positive semidefinite operators under linear output mappings. Despite these differences, both settings share the same abstract operator-theoretic structure: observable quantities are generated through operator-induced structure rather than through direct access to latent states. Within this perspective, representation transfer and knowledge distillation can also be interpreted geometrically. Within PPS, projection-mediated observability is treated as an explicit mathematical structure rather than as a purely metaphorical analogy between domains.

Given teacher and student observation operators $\Pi_{T}$ and $\Pi_{S}$ , a transfer map

$$\Phi :H_{T}\to H_{S}$$

approximately preserves observable geometry when

(6) $$\Phi \Pi_{T}\approx \Pi_{S}\Phi .$$

In the present formulation, Φ is assumed to be a linear transfer map acting on the latent representation space. This formulation is motivated by approximate intertwining relations commonly appearing in operator-theoretic alignment and representation-transfer settings. Equation (6) interprets distillation as approximate consistency between induced observation geometries, rather than solely as output-level distribution matching [27,28,29,30,31]. A central implication of PPS concerns the structural limits of output-based interpretability. Since observables generated through Equation (2) are invariant under perturbations contained in ker(Π) , they depend only on the equivalence classes induced by Equation (3) and represented by the quotient structure Equation (4). Latent components lying in ker(Π) cannot, in general, be recovered from observables alone. From this perspective, limitations of interpretability are not merely algorithmic, but geometric and operator-induced.

The PPS framework is conceptually related to several existing research directions. Information bottleneck formulations characterize information compression through mutual-information constraints [8,9,10,11,32], whereas PPS characterizes observability through operator-induced geometry on latent spaces. Similarly, concept bottleneck models and representation-constrained learning methods may be interpreted as imposing explicit structural constraints on induced observable subspaces [33,34,35,36]. PPS also exhibits structural parallels with observability theory in control systems [37], although the present framework focuses on projection-induced accessibility in representation spaces rather than dynamical state reconstruction.

To evaluate the PPS formulation empirically, we construct controlled latent-space experiments that directly examine projection-induced observability. The experiments verify three central predictions of the framework: (i) observables generated through Equation (2) remain invariant under perturbations confined to ker(Π) , (ii) the rank structure of Π governs effective observable dimensionality and predictive behavior, and (iii) attribution methods inherit structural limitations induced by the observation geometry. These experiments are designed primarily as theory-validation studies intended to isolate projection-mediated observability effects rather than as benchmark-oriented evaluations of predictive performance. Accordingly, the goal of the empirical analysis is not to establish state-of-the-art performance on large-scale datasets, but to directly test whether the structural predictions of PPS emerge under controlled experimental conditions.

The primary contribution of this work is not the introduction of new operator-theoretic machinery. While the mathematical ingredients of PPS, including positive semidefinite operators, kernels, quotient spaces, and spectral decompositions, are classical, the central novelty lies in identifying and formalizing an operator-induced observability structure that has not been explicitly characterized in existing representation-learning frameworks.

The central novelty lies in explicitly formulating observability as an operator-induced equivalence structure governing representation accessibility. In particular, PPS identifies the quotient geometry H/ker(Π) as the fundamental object underlying observable representations and provides a unified geometric framework connecting observability, representation transfer, and interpretability.

The resulting framework provides a mathematically explicit perspective on observability across projection-mediated representation systems. The main contributions of this paper are summarized as follows:

1. We introduce PPS, an operator-theoretic framework that formalizes observability in representation learning as an operator-induced geometric structure.
2. We identify operator-induced observability equivalence classes as a fundamental object governing representation accessibility and formalize them through the quotient geometry H/ker(Π) .
3. We provide a unified geometric interpretation of representation transfer, knowledge distillation, and output-based interpretability through operator-induced observability.
4. We establish the role of the spectral structure of positive semidefinite observation operators in determining effective observable dimensions and accessibility.
5. We provide empirical analyses validating key theoretical predictions of PPS, including kernel-invariant observability, projection-induced attribution gaps, and rank-controlled observable geometry.

## 2. Operator-Theoretic Framework of Observability

This section introduces Platonic Projection Structures (PPS) as an operator-theoretic framework for analyzing projection-mediated observability in representation learning systems. Rather than treating observable quantities as direct reflections of latent states, PPS models observation as a geometric process induced through a self-adjoint positive semidefinite operator acting on a latent Hilbert space.

### 2.1. Platonic Projection Structures

Let H denote a real or complex Hilbert space of latent representations. Within the PPS framework introduced in Equation (1), observability is induced through an observation operator Π:H→H, which generates observable quantities through the quadratic functional defined in Equation (2). We distinguish two regimes of observation:

1. Strict projection operators satisfying Equation (5);
2. Generalized positive semidefinite operators satisfying Π⪰0.

Idealized quantum measurements naturally belong to the first regime [21,22], whereas representation learning systems are generally associated with the second. Despite this distinction, both settings share the same structural mechanism: observable quantities are generated through operator-induced structure rather than through direct access to latent states. The observation operator is assumed to satisfy

$$\Pi^{†}=\Pi ,\;\langle v,\Pi v\rangle \geq 0\;\forall v\in H,$$

ensuring that the observable functional in Equation (2) is well-defined and non-negative. Under PPS, the operator Π becomes the primitive object governing observability. Observable structure is therefore determined not by latent representations themselves, but by the geometry induced through the observation operator.

### 2.2. Observable Geometry and Quotient Structure

Throughout this paper, the term “observable geometry” refers to the quotient-semimetric structure induced by Π through the equivalence relation in Equation (3) and the semi-inner product defined in Equation (7). A central consequence of PPS is that observability is defined only up to the equivalence relation introduced in Equation (3). Two latent states differing only by a component contained in ker(Π) are observationally indistinguishable under Equation (2). Accordingly, the effective observable space is not the ambient latent space H itself, but the quotient space H/ker(Π) defined in Equation (4), a standard construction in operator theory [12,13]. This quotient space represents equivalence classes of latent states that generate identical observables under the induced observation process. The operator Π further induces the bilinear form

(7) $${\langle v,w\rangle }_{\Pi }:=\langle v,\Pi w\rangle ,$$

which defines a semi-inner product structure on H . Since directions contained in ker(Π) contribute no observable signal, the form in Equation (7) becomes non-degenerate only after quotienting by ker(Π) . The quotient geometry in Equation (4) therefore defines the effective observable geometry induced by the observation operator [12,13]. Under this viewpoint, observability becomes a structural property of induced operator geometry rather than a property of individual latent vectors.

### 2.3. Spectral Structure of Observability

Building on classical spectral methods [15,16,17,18,19], the PPS framework naturally admits a spectral interpretation of observability. Let

(8) $$\Pi =\sum_{i}\lambda_{i}e_{i}e_{i}^{*}$$

denote the spectral decomposition of the observation operator. The eigenvalues $\lambda_{i}$ determine the degree of observability associated with each latent direction:

$$\lambda_{i}\gg 0\Rightarrow \mathrm{strongly}\;\mathrm{observable}\;\mathrm{direction},$$

$$\lambda_{i}\approx 0\Rightarrow \mathrm{weakly}\;\mathrm{observable}\;\mathrm{direction},$$

$$\lambda_{i}=0\Rightarrow e_{i}\in \ker\left(\Pi \right)\Rightarrow \mathrm{structurally}\;\mathrm{unobservable}\;\mathrm{direction}.$$

Accordingly, the effective observable dimension is governed by

(9) rank(Π).

Equation (9) implies that observable dimensionality depends not on the ambient latent dimension itself, but on the spectral structure of the observation operator [18,38]. From an information-theoretic perspective, the eigenspectrum in Equation (8) characterizes the relative accessibility of latent information under the induced observation process [8,20,32]. Large eigenvalues correspond to latent directions strongly represented in observable quantities, whereas directions associated with vanishing eigenvalues become inaccessible through Equation (2).

### 2.4. Structural Factorization of Observation

The observation process is induced by PPS factors naturally through the quotient geometry defined in Equation (4) [12,13]. Specifically, the observable functional in Equation (2) admits the factorization

(10) $$H\overset{\;q\;}{\to }H/\ker\left(\Pi \right)\overset{\;\tilde{O}\;}{\to }R,$$

where *q* denotes the canonical quotient map. Equation (10) formalizes a central implication of PPS: observable quantities do not uniquely determine latent states, but only equivalence classes induced by the observation geometry. Consequently, latent components lying in ker(Π) are structurally inaccessible from observables generated through Equation (2). This limitation is therefore geometric rather than merely algorithmic. From an information-geometric viewpoint, the quotient structure in Equation (4) may be interpreted as the effective observable information geometry induced by the observation operator [3,4,5,6]. The observation process therefore acts as a structured projection mechanism that compresses latent information according to the spectral accessibility encoded in Equation (8) [8,10,11]. Overall, PPS provides a unified operator-theoretic framework in which observability, representation accessibility, and projection-induced information loss are described through induced observable geometry [1,14,20].

### 2.5. Mathematical Setting and Assumptions

The PPS framework is formulated in a Hilbert space setting in order to provide a general operator-theoretic description of observability. Throughout the theoretical development, we assume that the observation operator Π:H→H is a bounded self-adjoint positive semidefinite operator. For the empirical studies presented in this paper, all latent representation spaces are finite-dimensional Euclidean spaces $R^{n}$ . In this setting, Π is represented by a symmetric positive semidefinite matrix, and its spectral decomposition is finite and exact. Consequently, the quotient geometry H/ker(Π) can be interpreted through standard finite-dimensional linear algebra. The Hilbert space formulation is retained primarily to emphasize the generality of the operator-theoretic perspective rather than to require infinite-dimensional analysis.

## 3. Projection-Mediated Observation Across Systems

This section shows that both quantum measurement and deep learning inference can be interpreted within the PPS framework as instances of projection-mediated observation [21,22,23,24]. The correspondence is structural rather than physical: in both settings, observable quantities are generated through operator-induced geometry acting on latent representation spaces [13,14,20].

### 3.1. Quantum Measurement as Projection-Induced Observation

Quantum measurement provides a canonical example of projection-mediated observability [21,22]. Throughout this paper, Π denotes a general observation operator in PPS, whereas $P_{m}$ refers specifically to a quantum measurement projector. A quantum system is represented by a normalized state vector $[|\psi \rangle \in H_{Q},\langle \psi |\psi \rangle =1,$ ] where $H_{Q}$ denotes a Hilbert space of admissible states. Measurement is described by a Hermitian operator admitting the spectral decomposition [13,21]

(11) $$M=\sum_{m}mP_{m},$$

where each $P_{m}$ is an orthogonal projection operator satisfying Equation (5). The probability of observing outcome *m* is given by the Born rule [21,22]

(12) $$p\left(m\right)=\langle \psi |P_{m}|\psi \rangle .$$

Equation (12) is structurally equivalent to the PPS observable functional defined in Equation (2) under the identification

$$v=|\psi \rangle ,\;\Pi =P_{m}.$$

From the PPS viewpoint, quantum measurement can therefore be interpreted as a projection-mediated observation process:

$$|\psi \rangle \to P_{m}\to p\left(m\right).$$

This formulation emphasizes that measurement accesses only the projected component of the quantum state. Information contained in complementary directions remains inaccessible under the induced observation process.

### 3.2. Deep Learning Inference as Operator-Induced Observation

Deep learning models equipped with linear output mappings can similarly be interpreted within the PPS framework as operator-mediated observation processes [1]. Let

(13) $$f_{\theta }=f_{L}\circ f_{L-1}\circ \cdots \circ f_{1}$$

denote a deep neural network, and let

$$z=f_{\theta }\left(x\right)\in H_{L}$$

represent the final latent representation. In standard architectures, outputs are generated through a linear readout

(14) y=Wz,

where *W* maps latent representations to output space. The corresponding quadratic observable becomes [14]

(15) $${∥y∥}^{2}=\langle z,W^{⊤}Wz\rangle .$$

Equation (15) naturally defines the positive semidefinite observation operator [14]

(16) $$\Pi :=W^{⊤}W.$$

Within architectures employing linear output mappings, Equation (16) shows that observable behavior is governed not solely by latent representations themselves, but also by the operator-induced geometry through which latent information becomes accessible [15,18,20]. The quadratic observable defined above should be understood as a canonical representation of the geometry induced by the linear readout operator. It does not aim to fully characterize nonlinear output mappings such as softmax-based classification. Such nonlinear observables lie outside the scope of the present formulation and constitute an important direction for future extensions.

Under PPS, deep learning inference can therefore be interpreted as

$$z\to \Pi \to {∥y∥}^{2},$$

which is structurally analogous to the projection-mediated process described by Equation (12). Importantly, PPS does not claim that neural networks are fundamentally quadratic systems. Rather, Equation (16) identifies a canonical observable geometry induced by the output map. Directions contained in ker(Π) remain inaccessible from observables generated through Equation (15). Consequently, latent representations are observable only up to the quotient geometry defined in Equation (4).

### 3.3. Structural Correspondence Between Quantum and Learning Systems

Both quantum systems and learning systems equipped with linear observation operators can be viewed through a common abstract structure [21,23,24,25] consisting of

1. A latent Hilbert space H ;
2. An observation operator Π ;
3. An induced observable generated through Equation (2).

Under this perspective, the two domains admit the formally parallel structure

$$\begin{array}{cc} \mathrm{Quantum}\;\mathrm{system}:\; & H_{Q},\;|\psi \rangle ,\;P_{m},\;p\left(m\right)=\langle \psi |P_{m}|\psi \rangle ,\\ \mathrm{Deep}\;\mathrm{learning}\;\mathrm{system}:\; & H_{L},\;z,\;W^{⊤}W,\;{∥y∥}^{2}=\langle z,W^{⊤}Wz\rangle . \end{array}$$

**Figure 1:**
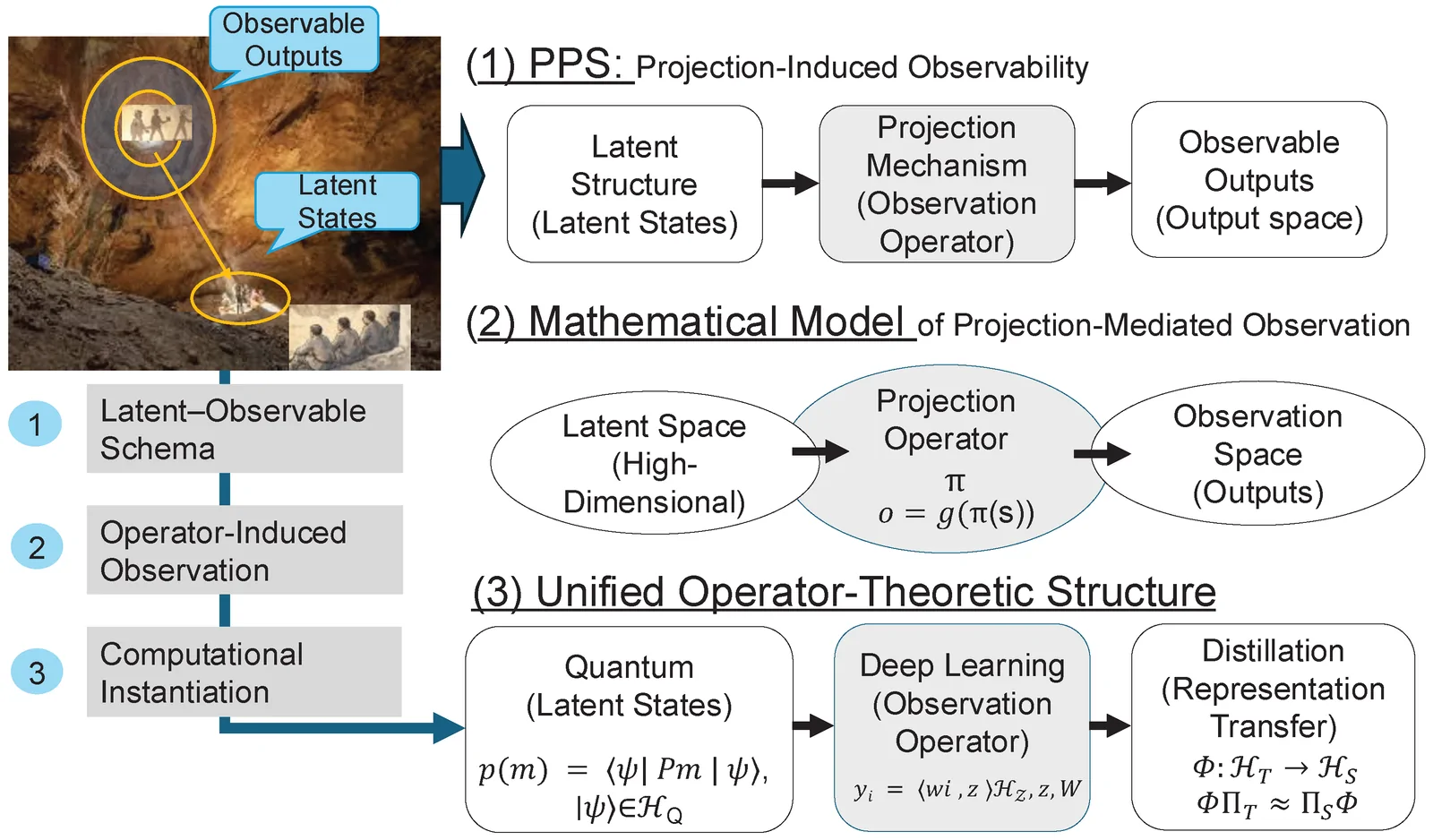
PPS framework as projection-induced observation geometry. Schematic illustration of projection-mediated observability under PPS. A latent state v∈H is mapped through a self-adjoint positive semidefinite operator Π to an observable quantity generated through Equation (2). Quantum systems correspond to projection operators satisfying Equation (5), whereas deep learning systems generally induce non-idempotent positive semidefinite operators such as Equation (16).

In both cases, observable quantities are induced through self-adjoint positive semidefinite operators acting on latent spaces [13,14]. The distinction lies not in the existence of projection-mediated observability itself, but in the algebraic structure of the corresponding operators. Quantum systems typically involve orthogonal projections satisfying Equation (5), whereas learning systems with linear output mappings induce non-idempotent positive semidefinite operators such as Equation (16). This correspondence should therefore be understood as operator-theoretic rather than physical. PPS does not identify neural inference with quantum dynamics, but instead provides a unified geometric language for describing observability across projection-mediated systems [23,24,26]. Figure 1 summarizes the resulting correspondence between these systems. PPS should therefore be understood as a simplified geometric characterization of observability under linear observation operators rather than as a complete description of modern neural-network architectures.

## 4. Information Geometry and Representation Transfer

Building on information theory, information bottleneck theory, information geometry, and representation transfer learning [8,20,27,32], this section develops an information-theoretic interpretation of PPS and introduces a geometric formulation of representation transfer. Within PPS, observable information is determined not only by latent representations themselves, but also by the observation geometry through which latent directions become accessible. Representation transfer can therefore be interpreted as approximate preservation of this induced observable geometry between latent spaces.

### 4.1. Information-Theoretic Interpretation of PPS

Motivated by information theory, information bottleneck theory, and information geometry [8,20,32], the PPS framework admits a natural interpretation from the perspective of information accessibility. Let v∈H be a latent representation, and consider observables generated through the quadratic functional defined in Equation (2). The observation operator determines which latent directions contribute to observable quantities. The spectral decomposition introduced in Equation (8) therefore characterizes the relative accessibility of latent information [8,20]. Large eigenvalues correspond to strongly observable latent directions, whereas vanishing eigenvalues identify directions that are completely unobservable. Accordingly, the effective observable dimension is governed by Equation (9), rather than by the ambient latent dimensionality itself. Observable quantities generated through Equation (2) depend only on information contained within the effective observable quotient defined in Equation (4).

### 4.2. Knowledge Distillation as Operator Alignment

Knowledge distillation can be interpreted as a geometric alignment problem between operator-induced observable structures [27,29,30,31]. Standard knowledge distillation is commonly formulated by minimizing the divergence between teacher and student output distributions [27]:

(17) $$L_{\mathrm{KD}}=D_{\mathrm{KL}}\left(p_{T}\|p_{S}\right),$$

where

$$p_{T}=\sigma \left(W_{T}z_{T}\right),\;p_{S}=\sigma \left(W_{S}z_{S}\right).$$

From the PPS viewpoint, Equation (17) aligns observable distributions in output space while leaving the geometry of the underlying observation operators implicit. Several extensions, including FitNets [28], RKD [29], CRD [30], and attention transfer [31], introduce increasingly structured alignment constraints between latent representations. Within PPS, these approaches may be viewed as introducing progressively richer forms of alignment that can be analyzed through the operator-alignment perspective. We associate the following positive semidefinite operators with teacher and student models:

(18) $$\Pi_{T}=W_{T}^{⊤}W_{T},\;\Pi_{S}=W_{S}^{⊤}W_{S}.$$

The corresponding quadratic observables become

(19) $$O_{T}\left(z_{T}\right)=\langle z_{T},\Pi_{T}z_{T}\rangle ,\;O_{S}\left(z_{S}\right)=\langle z_{S},\Pi_{S}z_{S}\rangle .$$

Equation (18) defines the induced observation geometries of the teacher and student systems.

### 4.3. Operator Consistency and Intertwining Relations

When the teacher and student latent spaces differ, we introduce a linear map

(20) $$\Phi :H_{T}\to H_{S}.$$

A natural notion of structural compatibility between teacher and student observation geometries is expressed through the approximate intertwining condition introduced previously in Equation (6). Equation (6) states that projecting before or after the transfer map yields approximately consistent observable structure. This condition expresses approximate preservation of observable structure under representation transfer. The corresponding commutative structure is represented by

$$\begin{array}{ccc} H_{T} & \overset{\;\Pi_{T}\;}{\to } & H_{T}\\ ↓\Phi & & ↓\Phi \\ H_{S} & \overset{\;\Pi_{S}\;}{\to } & H_{S} \end{array}$$

Under exact commutativity, observable quotient structures induced by Equation (4) are preserved under the transfer map. Approximate commutativity therefore provides a geometric notion of observable consistency between latent representation systems. To empirically examine this interpretation, we compare standard KL-based distillation with a PPS-regularized variant explicitly incorporating the operator-consistency objective

(21) $$L_{\mathrm{PPS}}={∥\Phi \Pi_{T}-\Pi_{S}\Phi ∥}_{F}^{2}.$$

### 4.4. Experimental Setup

To evaluate the PPS formulation in a practical representation-learning setting, we conducted a knowledge-distillation experiment on the CIFAR-10 dataset. The teacher model was a ResNet-18 classifier, while the student model consisted of a lightweight convolutional neural network with two convolutional blocks followed by two fully connected layers. Both models were trained on CIFAR-10 using the Adam optimizer with a learning rate of $10^{-3}$ and a batch size of 128. For reproducibility, all experiments were performed using a fixed random seed of 42. The teacher model was trained for 10 epochs. Student models were trained under two conditions: (1) standard knowledge distillation using KL-divergence and (2) PPS-aware distillation using KL-divergence together with the PPS commutativity regularization term defined in Equation (21). The distillation temperature was set to T=4.0 , and the PPS regularization coefficient was set to λ=0.5 . The observation operators were constructed from the final linear layers of the teacher and student networks as

$$\Pi_{T}=W_{T}^{⊤}W_{T},\;\Pi_{S}=W_{S}^{⊤}W_{S},$$

where $W_{T}$ and $W_{S}$ denote the corresponding output-layer weight matrices. The transfer map $\Phi :H_{T}\to H_{S}$ was implemented as a bias-free trainable linear transformation with $\Phi \in R^{d_{S}\times d_{T}}$ , where $d_{T}$ and $d_{S}$ denote the dimensions of the teacher and student penultimate feature spaces, respectively. For empirical analyses involving spectral quantities, numerical rank was computed using an eigenvalue threshold of $\varepsilon =10^{-6}$ , with eigenvalues below this threshold treated as numerically zero. The experiment was introduced specifically to evaluate whether the PPS formulation remains meaningful when applied to learned neural-network representations rather than synthetic latent-space constructions.

The primary objective of this experiment is not to establish superior predictive performance, but to evaluate whether operator-level consistency can be introduced as an explicit optimization target within representation-transfer frameworks. Whether reduced operator inconsistency leads to improved transferability, robustness, interpretability, or performance on unseen tasks remains an important open question for future investigation.

**Figure 2:**
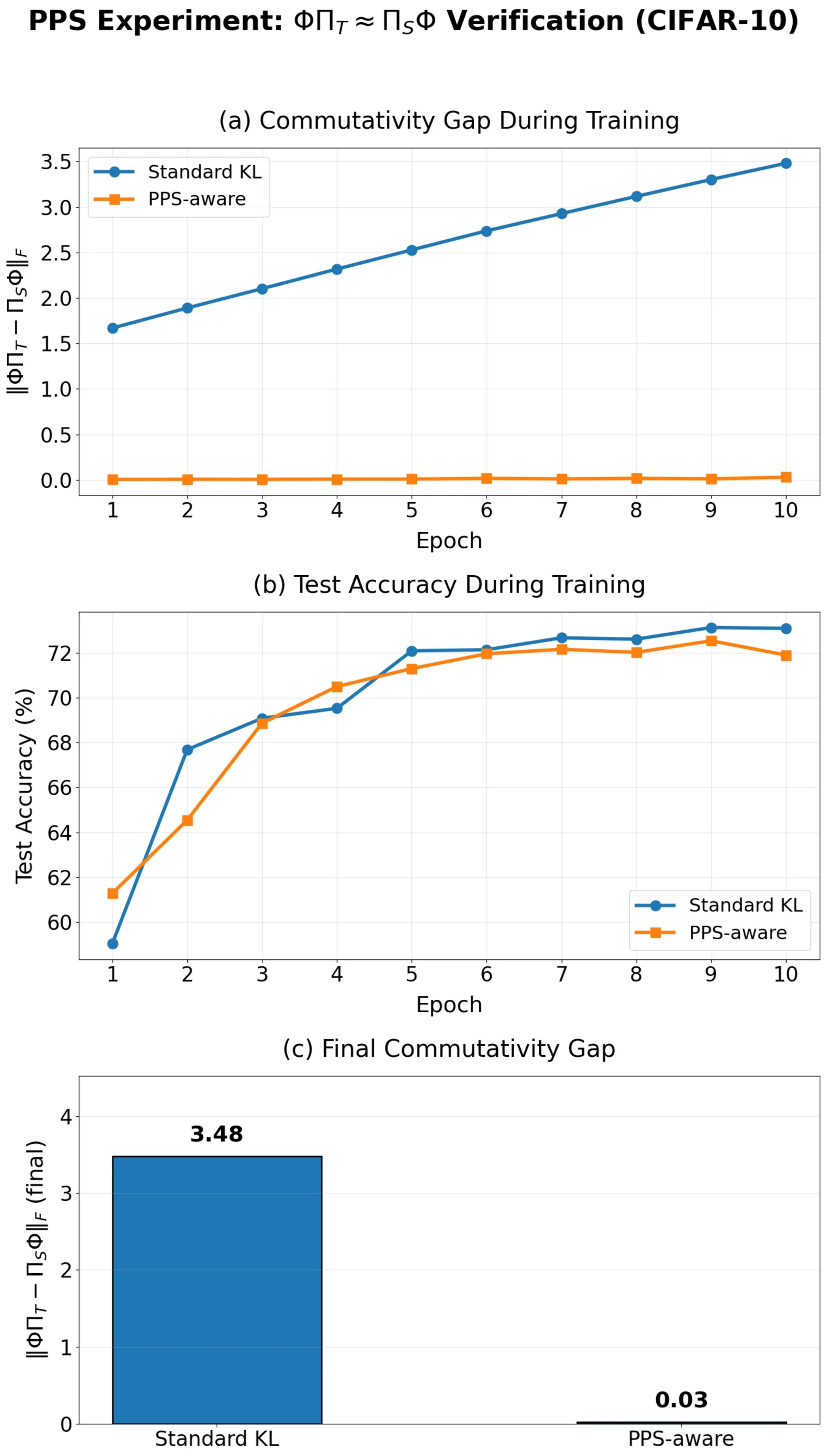
Operator-consistent knowledge distillation under PPS. Comparison between standard KL-based distillation and PPS-regularized distillation on CIFAR-10. The PPS formulation explicitly introduces the operator-consistency objective defined in Equation (21). (**a**) Evolution of the commutativity gap during training. (**b**) Test accuracy over epochs. (**c**) Final operator inconsistency after training. The results indicate that operator-level consistency can be introduced as an explicit optimization objective without degrading predictive performance in this setting.

Figure 2 summarizes the comparison between standard KL-based distillation and PPS-regularized distillation. Panel (a) shows the evolution of the commutativity gap induced by Equation (21), panel (b) reports predictive accuracy, and panel (c) shows the final operator inconsistency after training. The PPS-regularized formulation consistently reduces operator inconsistency between teacher and student systems while maintaining comparable predictive performance [27,29,30].

These results demonstrate that operator-level consistency can be measured and explicitly optimized within a standard distillation framework while maintaining comparable predictive performance. The findings suggest that observable geometry may provide a complementary perspective on representation transfer beyond output-level distribution matching, thereby motivating future investigation of transferability, robustness, and interpretability from an operator-theoretic viewpoint. Within PPS, the primary object of analysis therefore becomes the geometry induced by the operators themselves rather than the output distributions alone. The code used in this study is available from the corresponding author upon reasonable request.

Rather than claiming universality, this experiment serves as a controlled illustration that operator-level geometric consistency can be introduced as an explicit and measurable quantity within standard representation-transfer pipelines.

## 5. Structural Limits of Output-Based Interpretability

This section analyzes the structural limits of output-based interpretability from the PPS perspective [2,3,20]. The central implication is that explanation methods operating on observable outputs cannot, in general, access the full latent representation space. Instead, they inherit the geometric constraints imposed by the observation operator. Under PPS, observables generated through Equation (2) depend only on the operator-induced observable geometry. Consequently, latent components contained in ker(Π) are structurally inaccessible to explanation methods derived solely from outputs. This limitation is not a failure of a particular attribution algorithm, but a consequence of projection-mediated observability itself.

### 5.1. Projection-Induced Observability Limits

**Figure 3:**
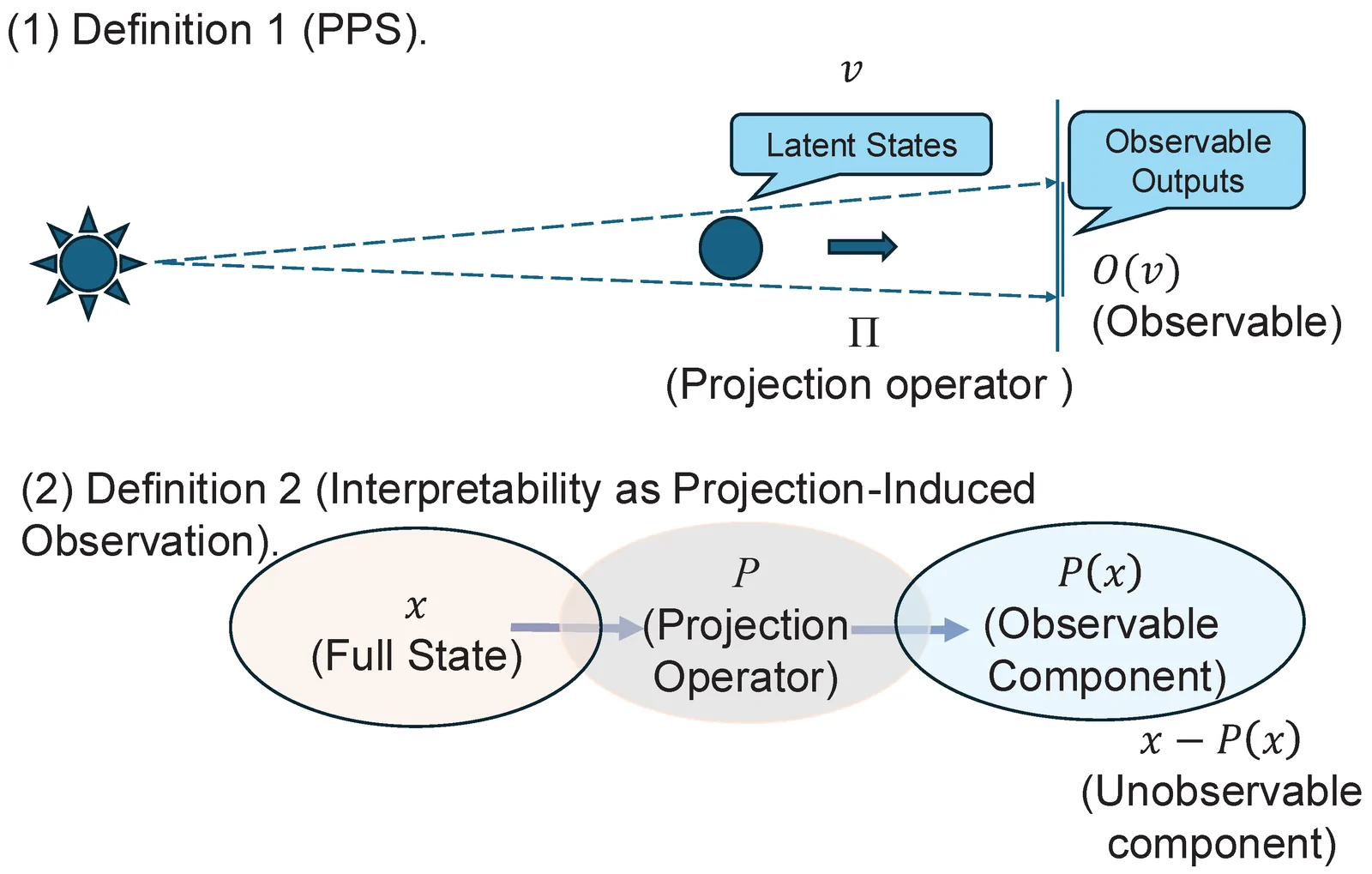
Conceptual illustration of observable and unobservable components under projection. The projection operator *P* maps a latent representation *x* to its observable component P(x) . The complementary component x−P(x) lies outside the observable subspace and is therefore inaccessible to output-based explanation methods.

To make this limitation explicit, we consider the strict projection regime introduced in Equation (5) [12,21]. Let P:H→H be an orthogonal projection operator. For a latent representation x∈H , the projection-induced decomposition is given by

(22) $$x=P\left(x\right)+\left(x-P(x)\right),$$

where P(x) is the observable component and x−P(x) lies in the complementary unobservable component. Equation (22) formalizes a key point: interpretability is not an intrinsic property of the latent representation *x* alone, but of the observation operator acting on it. In this regime, output-based explanations can access only the component preserved by *P*. Latent directions outside range(P) do not contribute to observable outputs and therefore cannot be recovered by explanation methods that operate only after projection. This projection-induced decomposition is illustrated conceptually in Figure 3.

### 5.2. Observational Equivalence Under Projection

The quotient geometry introduced in Equation (4) implies that distinct latent states may be observationally indistinguishable. The following proposition formalizes this observational equivalence. The observable equivalence structure is determined by the projection operator itself rather than by the scalar functional O(v) . The latter serves only as a low-dimensional summary statistic of the projected representation Πv and does not determine the full observable geometry.

**Proposition 1** 
(Observable Equivalence Under Projection). *Let the observable functional be defined as in Equation *(2)*, where *Π *is a self-adjoint positive semidefinite operator. If $v_{1}{∼}_{\Pi }v_{2}$ under Equation *(3)*, then*

(23) $$O\left(v_{1}\right)=O\left(v_{2}\right).$$

*Consequently, any explanation method that operates solely on observables generated through Equation *(2) *cannot distinguish between latent states belonging to the same equivalence class induced by ker(Π) .*

**Proof.**  Let $u=v_{1}-v_{2}$ with u∈ker(Π) . Then Πu=0 , and hence $\Pi v_{1}=\Pi \left(v_{2}+u\right)=\Pi v_{2}$ . Substituting this into Equation (2) yields Equation (23). Thus, observables cannot distinguish latent states differing only within ker(Π) . □

Although algebraically straightforward, Proposition 1 formalizes observational indistinguishability as a quotient-geometric property induced by the observation operator, providing the conceptual basis for the PPS framework.

**Corollary 1** 
(Non-Identifiability of Latent Structure). *Under PPS, latent representations are identifiable only up to the quotient geometry defined in Equation *(4)*. In particular, if dim(ker(Π))>0 , then the mapping from latent states to observables is non-injective.*

This result shows that output-based interpretability methods cannot reconstruct the full latent representation space. They can only characterize equivalence classes visible through the induced observation geometry.

### 5.3. Structural Interpretability and Attribution Limits

**Figure 4:**
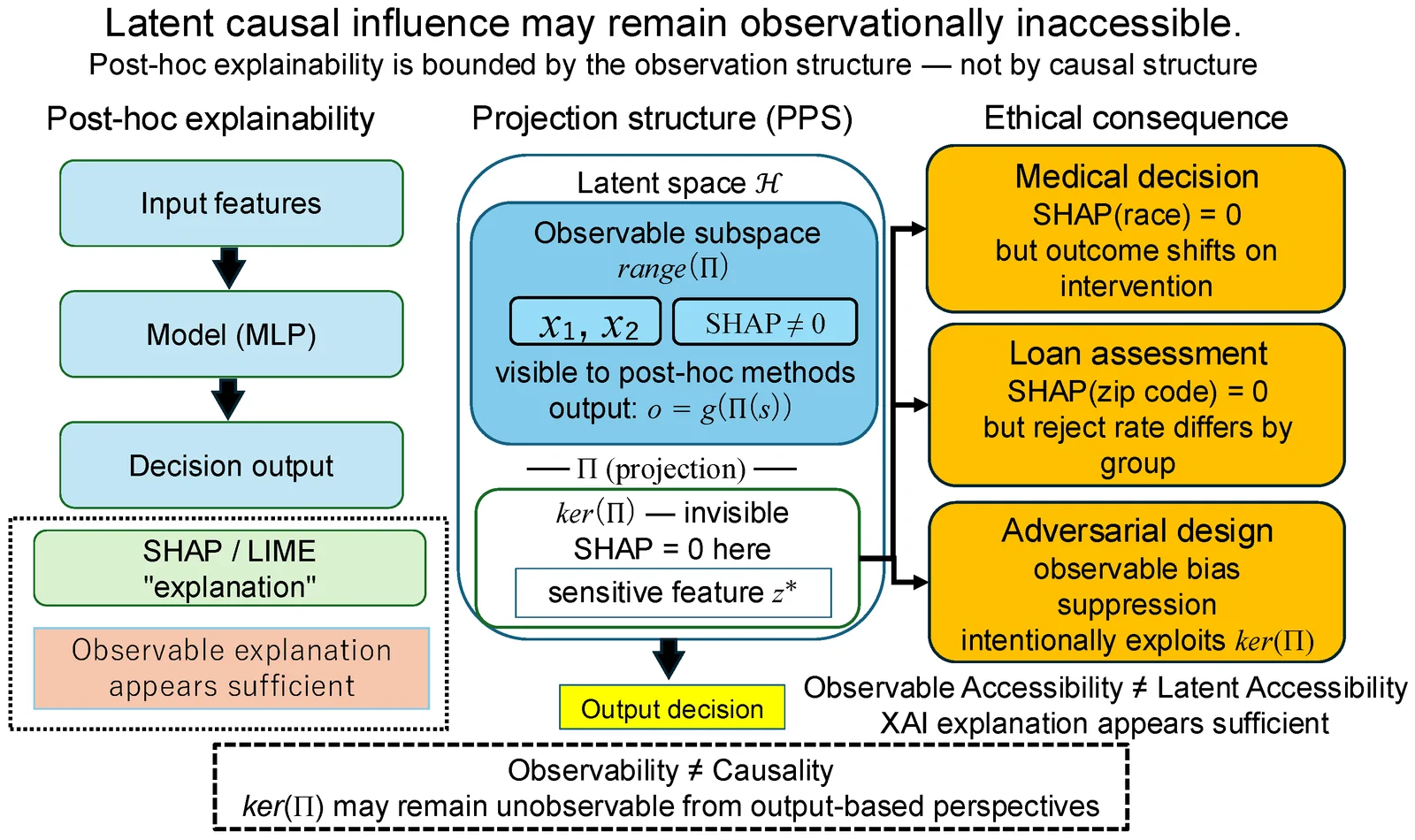
Structural limitations of post hoc explainability under PPS. Post hoc explanation methods operate only on the observable subspace induced by the observation operator. Latent components weakly represented within this observable geometry may remain inaccessible to output-based attribution methods such as SHAP, LIME, and GradCAM. Consequently, output-based explanations characterize only the observable quotient structure defined in Equation (4), rather than the full latent representation space.

PPS does not imply that post hoc attribution methods are invalid. Rather, the framework suggests that such methods are structurally bounded by the induced observation geometry through which observable quantities become accessible. The PPS framework provides a structural interpretation of post hoc interpretability methods such as SHAP [4], LIME [7], GradCAM [5], and integrated gradients [6]. These methods operate on outputs, activations, or gradients that are already constrained by the observation geometry. Therefore, they characterize only the observable component of the system, not the full latent structure. If a latent factor is encoded in a direction that lies in or near ker(Π) , its contribution to observables generated through Equation (2) is suppressed. As a result, attribution methods may assign negligible importance to a latent component even when that component remains meaningful within the pre-projection latent process. This distinction is important because observational sensitivity and intervention sensitivity need not coincide [36,39]. A latent component may be unobservable from the output perspective while still affecting downstream behavior through transformations that occur before projection. Thus, absence of attribution should not be interpreted as absence of latent relevance. Figure 4 summarizes this structural limitation of post hoc explanation.

**Figure 5:**
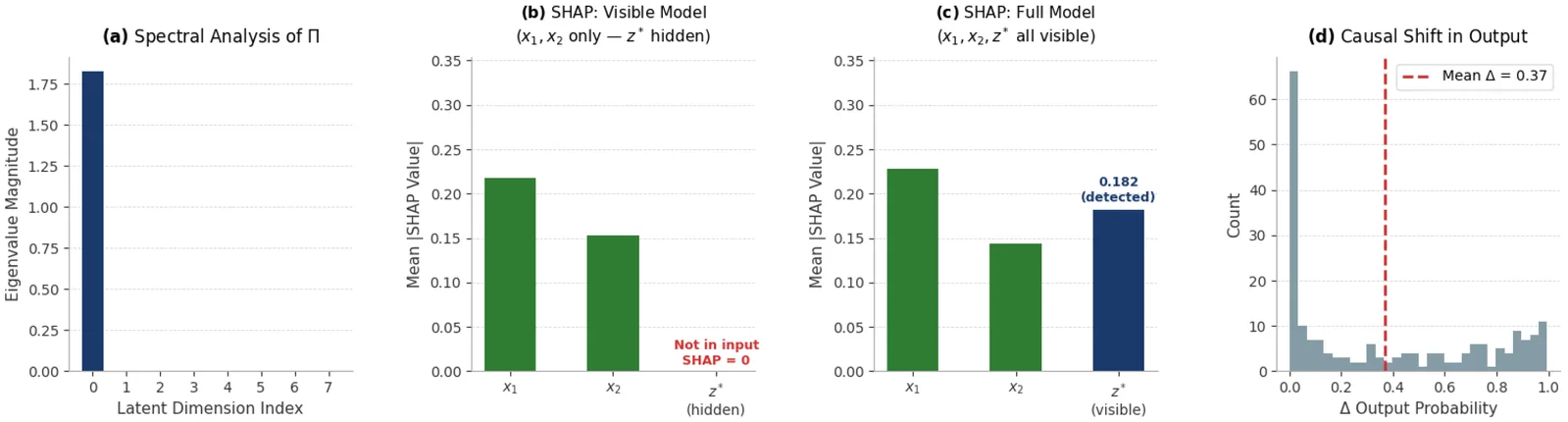
Empirical illustration of the observability–sensitivity gap under PPS. (**a**) Spectral structure of the observation operator showing a non-trivial kernel. (**b**) When the latent variable lies outside the observable subspace, SHAP attribution becomes negligible. (**c**) When the same variable is included in the observable representation, attribution becomes non-zero. (**d**) Latent intervention prior to projection induces measurable output variation despite negligible attribution. Together, these results show that attribution methods inherit structural limitations imposed by projection-mediated observability.

To illustrate this phenomenon empirically, we consider a controlled synthetic setting in which a latent variable $z^{*}$ is placed outside the observable subspace. When $z^{*}$ is excluded from the observable representation, SHAP assigns negligible attribution to it. When the same variable is explicitly included in the observable representation, the attribution becomes non-zero. This change reflects not a change in the underlying latent mechanism, but a change in the accessible observation geometry [14,20]. Importantly, the intervention in this experiment is applied before projection. Therefore, the experiment does not contradict Proposition 1, which concerns invariance under perturbations confined to ker(Π) after the observation geometry has been fixed. Instead, it demonstrates a separation between latent-space sensitivity and output-based attribution [36,39]. This observability–sensitivity gap is illustrated in Figure 5.

### 5.4. Toward Structural Accountability

Projection-induced observability limits suggest that output transparency alone is insufficient for characterizing the behavior of representation learning systems. This motivates a shift from output-centric explanation toward structural analysis of the observation operator itself. Within PPS, *structural interpretability* means explicitly characterizing the operator geometry that determines what can become observable. Correspondingly, *structural accountability* concerns whether the observation operator preserves, suppresses, or distorts application-relevant latent directions. This perspective leads to several structural questions:

- Which subspaces are preserved within range(Π) ?
- Which latent directions are eliminated by ker(Π) ?
- How does the spectral structure in Equation (8) regulate representational accessibility?
- How does the effective observable dimension in Equation (9) constrain explanation and prediction?

The PPS framework therefore suggests that interpretability constraints can be imposed directly at the operator level. Representative examples include spectral constraints controlling eigenvalue decay, subspace constraints enforcing preservation of designated latent directions, and operator regularization penalizing suppression of application-relevant components. Existing approaches can be reinterpreted in this language. Concept Bottleneck Models [33] impose explicit constraints on intermediate observable subspaces, while invariance-based learning methods may be viewed as inducing constraints on observation geometry [35,36]. PPS provides a unified formulation in which these methods correspond to different ways of shaping the operator that governs observability. From this perspective, interpretability is not merely a property of explanations produced after inference. Rather, it is a property of the observation geometry that determines what can become visible in the first place.

## 6. Empirical Verification of PPS

This section presents controlled experiments designed to directly evaluate central predictions of the PPS framework. Unlike conventional performance-oriented evaluations, these experiments are intentionally constructed as operator-level theory-validation studies that isolate projection-induced observability effects predicted by PPS.

The experiments focus on three theoretical consequences derived from the operator-theoretic formulation developed in previous sections:

1. Invariance of observables under perturbations confined to ker(Π) ;
2. Dependence of observable behavior on the rank and spectral structure of the observation operator;
3. Approximate preservation of observable geometry through operator-consistent representation transfer.

Accordingly, the empirical objective of this section is not to establish state-of-the-art performance on large-scale benchmarks, but rather to examine whether the theoretical predictions of PPS emerge under controlled experimental conditions.

### 6.1. Kernel-Invariant Observability

We first evaluate the PPS prediction that observables remain invariant under perturbations confined to the kernel of the observation operator [12,13]. Consider a latent representation space $H≅R^{D}$ equipped with a positive semidefinite observation operator Π⪰0 of rank R<D. The latent representation is explicitly decomposed into observable and structurally unobservable components:

(24) $$v=v_{\mathrm{obs}}+v_{\ker},$$

where

$$v_{\mathrm{obs}}\in \mathrm{range}\left(\Pi \right),\;v_{\ker}\in \ker\left(\Pi \right).$$

Observables are generated through the PPS quadratic functional defined previously in Equation (2). According to Proposition 1, perturbations restricted to ker(Π) should leave observables invariant, whereas perturbations within range(Π) should induce systematic observable variation. To verify this prediction directly, we constructed an 8-dimensional latent space with an observation operator of rank 4. The eigenspectrum therefore consists of four observable modes and four kernel modes. Two perturbation regimes were evaluated:

1. **Kernel perturbation:**
(25) $$v\to v+\delta_{\ker},\;\delta_{\ker}\in \ker\left(\Pi \right),$$
2. **Observable perturbation:**
(26) $$v\to v+\delta_{\mathrm{obs}},\;\delta_{\mathrm{obs}}\in \mathrm{range}\left(\Pi \right),$$

**Figure 6:**
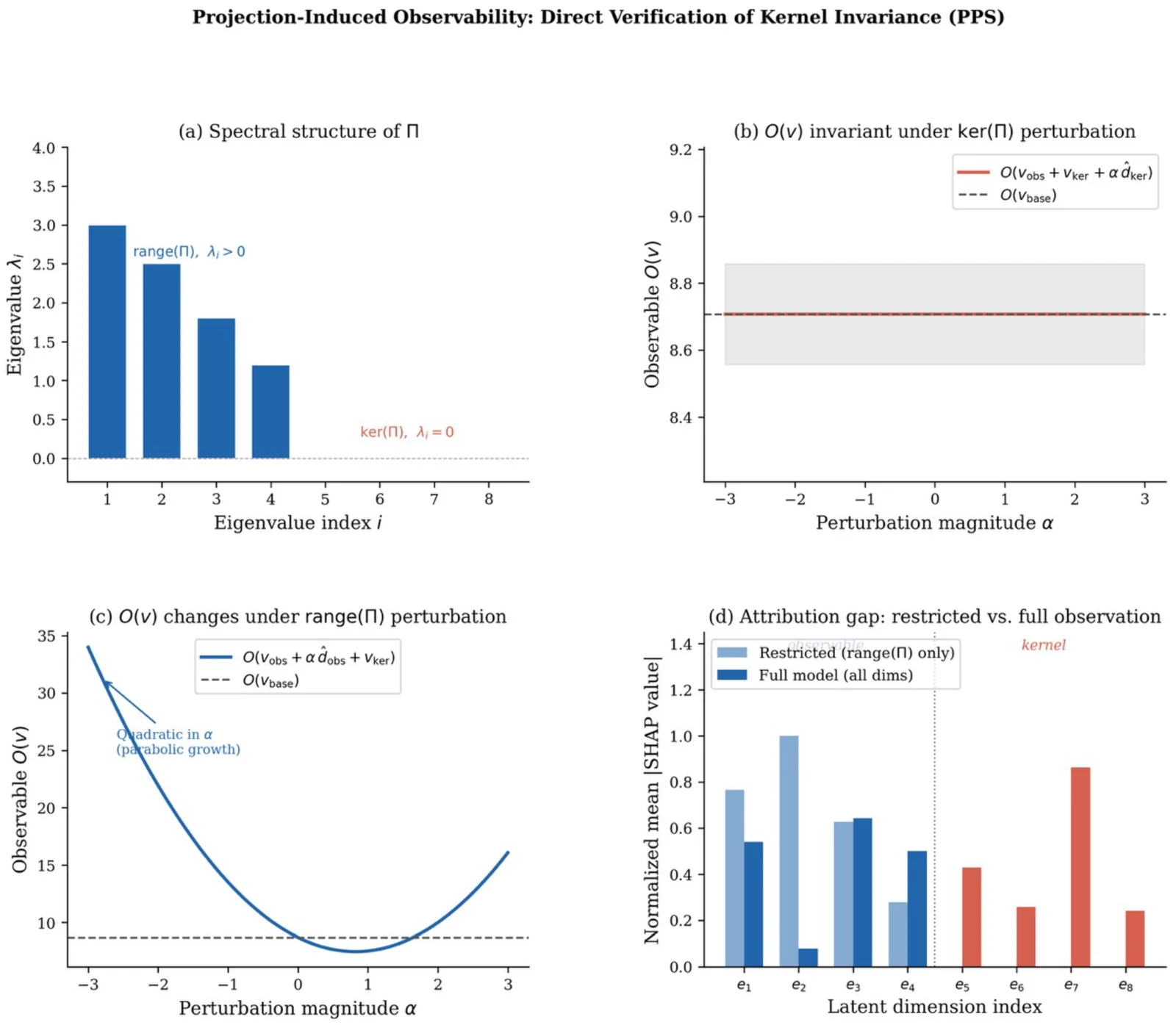
Direct verification of kernel-invariant observability under PPS. (**a**) Eigenvalue spectrum of the observation operator showing decomposition into observable and kernel directions. (**b**) Observable quantities remain invariant under perturbations confined to ker(Π) , directly validating Proposition 1. (**c**) Perturbations within range(Π) induce systematic observable variation. (**d**) SHAP attribution comparison between restricted and unrestricted models. Restricted models assign zero attribution to kernel dimensions, whereas unrestricted models do not.

Figure 6a shows the eigenspectrum of the observation operator, separating observable and kernel directions. Figure 6b demonstrates that perturbations generated through Equation (25) leave observables numerically invariant up to machine precision. In contrast, Figure 6c shows that perturbations generated through Equation (26) induce substantial observable variation with approximately quadratic dependence on perturbation magnitude. This provides a direct empirical visualization of the PPS prediction that latent directions contained in ker(Π) are structurally inaccessible under the induced observation geometry. Finally, Figure 6d compares SHAP attribution behavior between restricted and unrestricted models. When the model is explicitly constrained to range(Π) , kernel dimensions receive zero attribution by construction. In contrast, unrestricted models distribute attribution across both observable and kernel components. This suggests that attribution methods may reflect representational leakage outside the intended observable subspace structure induced by the operator.

These attribution experiments are intended as illustrative demonstrations of projection-induced observability limits rather than benchmark evaluations of attribution performance. The objective is to visualize the qualitative effect predicted by PPS, namely that attribution methods can only explain components that remain accessible through the induced observation geometry. Accordingly, statistical significance testing across multiple random seeds was not the primary focus of these controlled theory-validation experiments.

### 6.2. Rank-Controlled Observable Geometry

We next investigate how the rank structure of the observation operator governs effective observable geometry [17,18,38]. Within PPS, the effective observable dimension is determined by Equation (9). Consequently, observable behavior should depend not on the ambient latent dimensionality itself, but on the rank and spectral structure of the observation operator. To evaluate this prediction, we generated a synthetic classification task in a 16-dimensional latent space where the true signal was confined to an intrinsic subspace of dimension $r^{*}=8.$ Observation operators $\Pi_{r}$ with varying rank r∈{1,…,16} were constructed by progressively truncating the eigenspectrum introduced in Equation (8). For each rank setting, we evaluated

1. Classification accuracy;
2. Attribution leakage into ker(Π) ;
3. Cumulative observable spectral energy.

For empirical analyses involving rank estimation, numerical rank was computed using an eigenvalue threshold of $\varepsilon =10^{-6}$ , with eigenvalues below this threshold treated as numerically zero.

**Figure 7:**
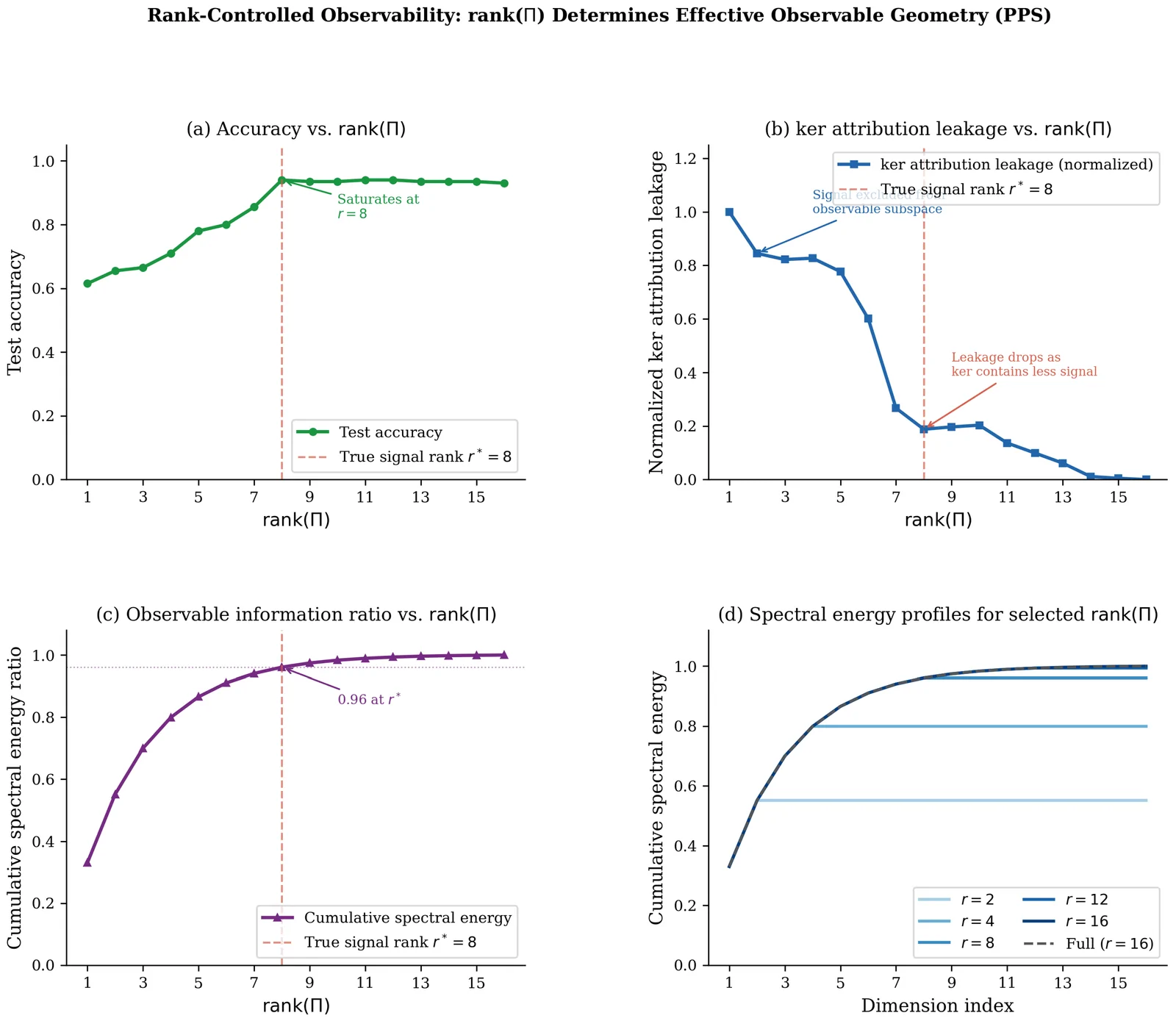
Rank-controlled observable geometry under PPS. (**a**) Classification accuracy as a function of rank(Π) . Performance increases systematically with observable dimension and saturates near the intrinsic signal rank. (**b**) Attribution leakage into ker(Π) decreases as the observable subspace expands. (**c**) Cumulative observable spectral-energy ratio captured by the induced observation geometry. (**d**) Spectral-energy accumulation profiles for multiple rank settings, illustrating how eigenspectrum truncation modifies effective observable structure.

Figure 7a shows that predictive performance increases systematically with rank(Π) and saturates near the intrinsic signal rank $r^{*}=8.$ Accuracy increases from approximately 0.61 at rank 1 to approximately 0.94 at rank 8, after which additional observable dimensions produce minimal improvement. Figure 7b shows the normalized kernel-attribution leakage ratio, defined as the proportion of attribution mass assigned to dimensions outside the observable subspace. Leakage decreases monotonically as rank(Π) increases, indicating that progressively more signal-bearing directions become observable. Figure 7c shows the cumulative observable spectral-energy ratio captured by the induced observation geometry. At $r^{*}=8$ , the observable subspace captures nearly all signal-associated spectral energy, consistent with the observed performance saturation. Finally, Figure 7d visualizes cumulative spectral-energy accumulation profiles for multiple rank settings, illustrating how eigenspectrum truncation modifies effective observable geometry. Collectively, these results support the PPS prediction that observable behavior is governed by the spectral structure of the observation operator rather than by latent dimensionality alone.

### 6.3. Operator-Consistent Knowledge Distillation

Finally, we evaluate the PPS interpretation of representation transfer introduced in Equation (6) [27,29,30]. The experiment compares standard KL-based knowledge distillation [27] with a PPS-regularized formulation incorporating the operator-consistency objective defined in Equation (21). A ResNet18 teacher model and a lightweight CNN student model were trained on CIFAR-10. The interspace map introduced in Equation (20) was implemented as a trainable linear projection jointly optimized with the student network.

Figure 2 summarizes the comparison between standard distillation and PPS-regularized distillation. Panel (a) shows the evolution of the commutativity gap induced by Equation (21), panel (b) reports predictive accuracy, and panel (c) shows the final operator inconsistency after training. The PPS-regularized formulation consistently reduces operator inconsistency between teacher and student systems while maintaining comparable predictive accuracy. Importantly, the improvement occurs at the level of induced observation geometry rather than solely at the level of output agreement. These results support the PPS interpretation that successful representation transfer may depend not only on observable output matching, but also on approximate compatibility between the corresponding observation operators. Rather than serving as a benchmark-oriented evaluation, the experiment provides a controlled empirical verification that operator-induced geometry can be treated as an explicit and measurable object within representation-transfer pipelines.

## 7. Discussion

The PPS framework provides an operator-theoretic perspective on observability in representation learning systems [1,12,13,14]. Rather than treating observable outputs as direct reflections of latent representations, PPS models observation as geometry induced through self-adjoint positive semidefinite operators acting on latent Hilbert spaces [12,14,20]. This viewpoint connects several previously separate research directions, including information bottleneck theory, observability theory, representation learning, and interpretability analysis [1,2,3,8,37].

### 7.1. Relation to Information Bottleneck and Representation Learning

PPS is conceptually related to the information bottleneck (IB) principle [8,10,11,32], which studies compressed representations through mutual-information constraints. Both frameworks emphasize that learned systems preserve only selected aspects of latent information. However, the two approaches differ fundamentally in formulation. Information bottleneck methods characterize compression probabilistically through random variables and mutual-information objectives [8,10,11,32], whereas PPS characterizes observability geometrically through operator-induced quotient geometry and spectral structure. Within PPS, information accessibility is governed by the quotient geometry introduced in Equation (4) and the eigenspectrum defined in Equation (8). Observable information therefore becomes a property of induced operator geometry rather than solely of probabilistic compression. This perspective also relates naturally to modern representation learning. Many learning systems implicitly construct observable subspaces through learned readout operators, as exemplified by Equation (16) [1,15]. From the PPS viewpoint, latent representations should therefore be interpreted together with the observation geometry through which they become accessible.

### 7.2. Relation to Observability Theory

PPS also exhibits structural parallels with classical observability theory in control systems [37]. Classical observability theory studies whether internal dynamical states can be reconstructed from observable outputs over time. By contrast, PPS focuses on structural accessibility induced by observation operators acting on latent representation spaces [12,13,14]. Despite this distinction, both share a common conceptual principle: observable behavior depends not only on latent states themselves, but also on the structure governing access to those states. Within PPS, this structure is encoded through the operator geometry induced by Π and the quotient structure defined in Equation (4).

### 7.3. Interpretability and Structural Accessibility

A central implication of PPS concerns the structural limits of output-based interpretability [2,3]. Since observables generated through Equation (2) are invariant under perturbations in ker(Π) , they depend only on the quotient structure defined in Equation (4). The experiments presented in Section 5 and Section 6 directly illustrate this phenomenon. Kernel-confined perturbations remain observationally invisible despite latent modification, while eigenspectrum truncation systematically alters attribution behavior and effective observable dimensionality. These results suggest that attribution maps and output-based explanations should not be interpreted as direct reconstructions of latent structure. Rather, they characterize only the observable geometry induced through the observation operator. From this viewpoint, interpretability limitations are not merely algorithmic deficiencies, but structural consequences of projection-mediated observability.

### 7.4. Relation to the Platonic Representation Hypothesis

Recent discussions surrounding the Platonic Representation Hypothesis [40] suggest that independently trained neural systems may converge toward structurally similar latent representations. PPS provides a complementary perspective on this idea. Rather than focusing solely on latent representation similarity, PPS emphasizes the geometry induced by observation operators acting on those representations. From this viewpoint, similarity between systems may arise not only from alignment of latent states themselves, but also from compatibility between induced observable geometries. The operator-consistency relation introduced in Equation (6) provides one possible formalization of this perspective. The knowledge-distillation experiments further suggest that approximate preservation of operator geometry may serve as a measurable notion of structural compatibility between representation systems [27,29,30,31]. More broadly, PPS suggests that observability may provide a useful geometric language for relating representation learning, interpretability, information accessibility, and projection-mediated inference across diverse systems [1,14,20]. In this context, the term “Platonic” is used structurally to emphasize the distinction between latent structure and observable projection, rather than to invoke a metaphysical claim.

### 7.5. Limitations and Future Work

Several limitations of the present work should be emphasized.

First, the current PPS formulation primarily analyzes static observation geometry. Extensions to temporally evolving operators and sequential systems remain future work.

Second, the empirical experiments are intentionally controlled and illustrative rather than benchmark-oriented. The primary goal is conceptual validation of projection-mediated observability rather than state-of-the-art predictive performance.

Third, the present framework focuses primarily on linear self-adjoint positive semidefinite observation operators [13,14]. Extensions to nonlinear operators, stochastic observation processes, non-self-adjoint operators, non-Hilbert latent geometries, and temporally evolving observability remain important directions for future work.

- Operator-constrained representation learning;
- Spectral regularization for interpretable latent geometry;
- Projection-aware explanation methods;
- Geometric formulations of transfer learning;
- Extensions to sequential and multi-modal systems.

While the present study focuses on controlled synthetic experiments and a CIFAR-10 distillation setting, broader empirical validation remains an important direction for future work. In particular, evaluating PPS on large-scale vision benchmarks, natural-language representation models, medical imaging datasets, and foundation-model representations would provide additional evidence regarding the practical applicability of the framework beyond the controlled settings considered here.

For a nonlinear readout

y=f(z),

a possible extension of PPS can be obtained through local linearization around a reference representation $z_{0}$ :

$$f\left(z\right)\approx f\left(z_{0}\right)+J_{f}\left(z_{0}\right)\left(z-z_{0}\right),$$

where $J_{f}\left(z_{0}\right)$ denotes the Jacobian of the readout. This induces a local observation operator

$$\Pi_{\mathrm{local}}=J_{f}{\left(z_{0}\right)}^{T}J_{f}\left(z_{0}\right).$$

Under this formulation, observability becomes state-dependent, yielding a local observable geometry that may vary across the representation manifold. Related approximations based on empirical Fisher information matrices may provide an alternative route toward nonlinear observability analysis. Experimental validation of Jacobian- or Fisher-based observation operators requires substantially different benchmark settings and is therefore left for future work.

Another important direction concerns attribution analysis under controlled latent factors and spurious correlations. Within PPS, explanatory signals associated with latent variables lying in ker(Π) are expected to be suppressed or absent from observable outputs, whereas observable latent directions may contribute strongly to attribution scores.

Empirical validation of this prediction on real-world datasets remains an important topic for future work. More broadly, future work should investigate whether PPS-based representations improve robustness under out-of-distribution conditions or facilitate transfer learning across related tasks. Such evaluations would provide further evidence regarding the practical consequences of operator consistency.

### 7.6. Relationship to Existing Frameworks

The relationship between PPS and information bottleneck methods has been discussed above. Here, we compare PPS more broadly with existing representation-analysis frameworks, including kernel PCA, canonical correlation analysis (CCA), and information bottleneck formulations. Although PPS shares certain structural similarities with these approaches, it addresses a fundamentally different question: which latent directions become observable under a given observation operator.

Kernel PCA and related spectral methods [15,17] analyze latent representations through spectral decomposition of kernel-induced operators. Their primary objective is dimensionality reduction and extraction of informative low-dimensional structure. In contrast, PPS does not seek dimensionality reduction itself. Rather, PPS characterizes which latent directions are observable under a given observation operator and formalizes observability through the quotient geometry H/ker(Π) .

Similarly, canonical correlation analysis (CCA) and representation-alignment methods [41] seek correlated subspaces between multiple representations. These approaches are widely used for comparing neural representations and analyzing transferability across models. PPS addresses a complementary problem: instead of maximizing correspondence between representations, it characterizes the observable geometry induced by the observation operator itself. From the PPS perspective, representation-alignment methods may be interpreted as implicitly constructing mappings between observable quotient structures.

Information bottleneck formulations [8,10] provide an information-theoretic framework in which representations are optimized through a trade-off between compression and predictive relevance. PPS differs in that it does not explicitly quantify information content through mutual information. Instead, it characterizes representation accessibility through operator-induced observability. In this sense, information bottleneck methods focus on how much information is retained, whereas PPS focuses on which latent directions can become observable.

These perspectives are therefore complementary rather than competing. Kernel methods, CCA, and information bottleneck approaches may all be viewed as constructing, constraining, or analyzing operators acting on latent representations. PPS provides an operator-theoretic framework for analyzing the observable geometry induced by such operators and the resulting limits of representation accessibility. Consequently, PPS should be viewed not as a replacement for these frameworks, but as an operator-theoretic perspective that clarifies the geometric conditions under which latent information becomes observable.

## 8. Conclusions

This paper introduced Platonic Projection Structures (PPS), an operator-theoretic framework for analyzing projection-mediated observability in representation learning systems. Within PPS, observable quantities are generated not through direct access to latent representations, but through geometry induced by self-adjoint positive semidefinite observation operators. The framework formalizes this perspective through the observable functional defined in Equation (2), the quotient structure introduced in Equation (4), and the spectral decomposition in Equation (8). From this viewpoint, observability becomes a structural property of induced operator geometry rather than a property of latent states themselves. The framework further provides a unified abstraction spanning quantum measurement, deep learning inference, representation transfer, and interpretability analysis [1,21,22,27]. Quantum systems and neural systems were shown to admit structurally parallel formulations as projection-mediated observation processes, despite substantial differences in physical interpretation [21,23,24]. PPS also provides a geometric interpretation of representation transfer through the operator-consistency relation introduced in Equation (6). The corresponding experiments demonstrated that operator-level consistency can be incorporated explicitly within knowledge-distillation pipelines while preserving predictive performance. A central implication of PPS concerns the structural limits of output-based interpretability. Because observable quantities depend only on the induced observable quotient geometry, attribution methods operating exclusively on outputs necessarily inherit constraints imposed by the observation operator itself. The controlled experiments presented in this work verified several central predictions of the framework, including

1. Kernel-invariant observability;
2. Rank-controlled observable geometry;
3. Operator-consistent representation transfer.

Collectively, these results support the PPS interpretation that observable behavior is governed by operator-induced geometry rather than by latent representations alone. More broadly, PPS suggests that observability may serve as a unifying geometric principle connecting representation learning, interpretability, information accessibility, and projection-mediated inference across diverse systems. Future work will investigate extensions to nonlinear operators, sequential systems, adaptive observation geometry, multi-modal architectures, and projection-aware interpretability methods. Ultimately, the PPS framework proposes a shift in perspective: rather than asking only what latent representations contain, one may instead ask what the induced observation geometry allows a system to observe.

## Data Availability

The synthetic data used in this study can be reproduced using the procedures described in the manuscript. The code used for the synthetic experiments, CIFAR-10 distillation experiments, and synthetic-data generation procedures are openly available in the GitHub repository at https://github.com/kazuoishii/Platonic-Projection-Structures (accessed on 28 June 2026).

## Abbreviations

The following abbreviations are used in this manuscript:

PPS	Platonic Projection Structures
KL	Kullback–Leibler
KD	Knowledge Distillation
IB	Information Bottleneck
CNN	Convolutional Neural Network
PSD	Positive Semidefinite
SHAP	SHapley Additive exPlanations
XAI	Explainable Artificial Intelligence
